# Significance of chromogranin A and synaptophysin in medullary thyroid carcinomas

**DOI:** 10.17305/bjbms.2020.5407

**Published:** 2021-10

**Authors:** Tatsuo Tomita

**Affiliations:** Department of Integrative Bioscience, Oregon Health and Science University, Portland, Oregon, United States

**Keywords:** Calcitonin, chromogranin A, immunohistochemistry, medullary thyroid carcinoma, multiple endocrine neoplasm, neuroendocrine tumors, synaptophysin, thyroid

## Abstract

Medullary thyroid carcinoma (MTC) is a rare thyroid carcinoma of C-cell deviation that produces and secretes calcitonin (CT) and chromogranin A (CgA) into the blood. Thus, CT and CgA are immunohistochemical and serum markers for MTCs. MTC occurs in sporadic and inheritable forms. The hallmark of inheritable cases in multiple endocrine neoplasm 2 (MEN2) is MTC. MEN2 cases represent 30% through germline RET proto-oncogene mutation and occur in younger ages involving bilateral thyroid lobes. Sporadic cases are 70% and occur in older ages. CgA and synaptophysin (SPY) are the two most widely used and reliable immunohistochemical markers for neuroendocrine tumors, including MTCs. This study aimed to detect different immunohistochemical staining patterns for CgA and SPY between non-symptomatic small lesions and invading larger aggressive tumors in both MEA2 cases and sporadic cases. There was different CgA and SPY immunostaining in MEA2 cases where small tumors (≤0.3 cm) were lesser immunostained for CgA and SPY, despite intense staining for CT, compared to the larger (≥0.5 cm) tumors, stronger immunostained for CgA. There was also different CgA and SPY immunohistochemical staining in sporadic cases between small lesions (≤0.5 cm) and larger tumors (≥1.0 cm). One small sporadic tumor (0.5 cm × 0.3 cm) was strongly and weakly, patchy stained for CgA and SPY, respectively, while larger sporadic tumors were diffusely and strongly stained for CgA and SPY. Therefore, stronger CgA and SPY immunostaining for larger tumors in both MEA2 and sporadic cases may be used as independent, aggressive immunohistochemical markers for MTCs.

## INTRODUCTION

Medullary thyroid carcinoma (MTC) is a relatively rare thyroid carcinoma of C-cell deviation, accounting for 2-3% of all thyroid cancers [1-3], at a lower frequency than the previously reported frequency of 3-12% [4,5]. Calcitonin (CT) producing C-cells comprise a minor component of the normal human thyroid gland, accounting for <0.1 % of the total thyroid epithelial cells [1]. C-cells are not evenly distributed but are restricted in the distribution to the junctions of the upper and middle third of the thyroid lobes along a hypothetical central axis, from which MTCs usually arise [1,3]. MTCs occur in sporadic cases (70%) and inheritable cases (30%) [4,5], and sporadic MTC occurs more frequently in the 4^th^-6^th^ decades while inheritable cases occur in younger ages of 2^nd^ and 4^th^ decades [1,3]. MTCs are the hallmark of the multiple endocrine neoplasm type 2 syndrome (MEN2) occurring almost in all cases (>95%), in the background of a gain-of-function germline mutation of *RET* proto-oncogene (10q11.2), which encodes a receptor tyrosine kinase [6-8]. MEN2 includes MEN2A, MEN2B, and familial MTC (FMTC) [1,4]. MEN2 presents clinically in earlier ages than the sporadic cases, MEN2A presenting at 25-35 years of age, and MEN2B presenting 10-20 years of age while FMTC occurs at 45-55 of age at about the same age of the sporadic cases [1,4]. MTCs have a propensity for regional lymph node metastasis, and the average 10-year survival for MTCs ranges from 61% to 75%, depending on the stages [3]. With serum CT immunoassay, the family members inherited with MEN2 are detected in the early stage with elevated serum CT levels, often before developing clinically detectable MTCs [9-11] and following a better prognosis with prophylactic thyroidectomy [9,10]. By immunohistochemical staining, MTCs are consistently positively stained for CT, chromogranin A (CgA), and carcinoembryonic antigen, and this study presents immunohistochemical staining patterns for CT, CgA, and synaptophysin (SPY) as the latter two are the most widely used and reliable markers for neuroendocrine cells and tumors [12]. CgA is widely distributed in neuroendocrine cells, including adrenal medulla, thyroid C-cells, intestinal neuroendocrine cells, parathyroid chief cells, anterior pituitary cells, pancreatic endocrine cells, and others [12,13]. CgA is not only a marker for neuroendocrine cells and it appears as a marker for biological malignancy in some endocrine tumors [14-16]. This study aimed to explore the possible immunohistochemical difference between the small tumors (≤0.5 cm) and larger tumors (≥1.0 cm) in sporadic cases, and a similar difference between small tumors containing *in-situ* lesion (≤0.3 cm) and grossly visible tumors (0.5-0.7 cm) in MEN2 cases [1,5].

## MATERIALS AND METHODS

All normal thyroid tissues and MTC tissues were collected at the University of Kansas Medical Center between 1975 and 2001. Five normal thyroids, nine sporadic MTCs, and four MTCs of the presumed MEN2 families were studied. Most of these cases were previously reported by us [17,18]. All normal thyroid and MTC tissues were routinely fixed in formalin and embedded in paraffin. The archival paraffin blocks were freshly sectioned, and deparaffinized sections were treated with antigen retrieval procedure using citrate buffer pH 6.2. The primary antibodies were as follows: Rabbit anti-CT at 1:20 (Signet Lab, Durham, MA), monoclonal anti-CgA at 1:100 (Dako, clone DAK-A3, Santa Clara, CA) at 1:100, and rabbit anti-SPY at 1:100 dilution (Cell Marque, Cat 336-76, Rocklin, CA). The staining procedure was the same as reported before [16-18]. Immunohistochemical staining was performed at each batch of 20 tissue sections to obtain good comparative staining. The normal thyroid gland and the adjacent normal thyroid gland were used as the internal control at +++ staining. The weaker staining was graded as ++ and +, respectively, and 0 for negative staining.

## RESULTS

**FIGURE 1:**
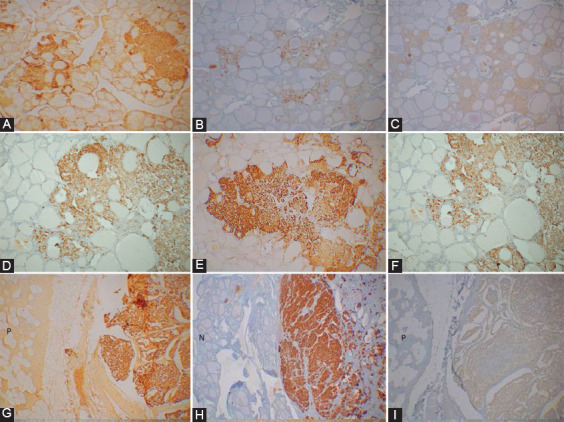
Presumed Multiple Endocrine Neoplasm 2 cases. Case. 4. A 36-year-old woman had bilateral MTCs at the upper one-third lobe, measuring 0.6 cm and 0.2 cm, respectively. The smaller tumor showed stronger granular immunostaining for CT (A) and weakly granular staining for chromogranin A (CgA) (B) and synaptophysin (SPY) (C). Case 3. A 28-year-old woman had 0.5 cm tumors at the right and left upper lobes, which were moderately to diffusely, strongly positive for CT (D) and CgA (E), and weakly diffusely positive for SPY (F). Case 2. A 26-year-old woman had a 0.7 cm MTC at the left upper lobe, which was diffusely, strongly positive for CT (G) and CgA (H), respectively, and weakly, diffusely positive for SPY (I). n---Normal thyroid gland, p---Normal parathyroid. A and D: CT; (B and E): CgA and C and F: SPY immunostained A, D, and G: CT, B, E, and H: CgA and C, F, and I: SPY immunostained.

**FIGURE 2:**
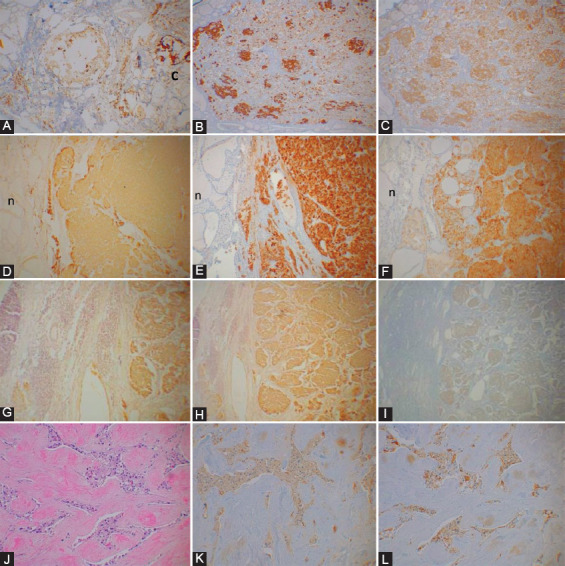
Sporadic cases. Case 4. A 42-year-old man underwent right thyroid lobectomy for papillary thyroid carcinoma and a small MTC (0.5 cm × 0.3 cm) was found in the lobectomy specimen. The tumor was partially, faintly positive for CT (about 70% of tumor cells) (A) and patchy, roundly strongly positive for chromogranin A (CgA) (about 10% of tumor cells) (B) and patchy, roundly weakly positive for synaptophysin (SPY) (about 10% of tumor cells) (C) with dystrophic calcification in the middle of the tumor, which was nonspecifically stained for CT, CgA, and SPY. Case 1. A 26-year-old man had a 2.5 x.1.8 x 1.4 cm tumor at the right lower lobe, which was diffusely, moderately stained for CT (D) and diffusely, strongly positive for CgA (E) and SPY (F), respectively. Case 6. A metastatic neck lymph node (L) from a 49-year-old woman was diffusely, moderately positive for CT (G) and CgA (H) and diffusely, weakly for SPY (I). Case 5. A 47-year-old man had diffuse metastasis in the femur, showing fibrous stroma embedded by trabecular tumor cell cords (H & E section, J), which were weakly positive for CT (K), CgA (L), and SPY, the latter staining was of the similar staining of that of CT (K) and CgA (L). L: Lymph node, n---Normal thyroid gland A, D, G and K: CT; B, E, I, and L: CgA and C, F, and I: SPY immunostained, J: H and E stained.

Among four presumed MEN family cases, the youngest was a 14-year-old boy, and the oldest was a 36-year-old woman (Table 1). All four cases involved both the right and left bilateral upper lobes by MTCs (Table 1). Case 1, the youngest, 14-year-old boy, a presumed MEN2B case underwent total prophylactic thyroidectomy without grossly identifiable tumors. There were bilateral multiple MTC lesions in the upper one-third of the thyroid lobe containing adjacent in-site lesions (Figure not shown). The second smallest tumor in Case 4 was a microscopic tumor, measuring 0.2 cm from the left lobe, spread along the interfollicular fine connective tissues containing lymphatic and blood vessels and was diffusely, moderately positive for CT in the main tumor and was weakly stained in the adjacent in-situ tumor for CgA and SPY (Table 1, Figure. 1 A-C). Case 3 contained bilateral 0.5 cm tumors, which were moderately positive for CT and moderately to strongly positive for CgA, and weakly to moderately positive for SPY, respectively (Table 1, Figure.1 D-F). Case 2 of a presumed MEN2A case, a 26-year-old man, contained multiple scattered microscopic MTC lesions, one from the left lobe measuring 0.7 cm, which was strongly immunostained for CT and CgA and weakly positive for SPY (Figure. 1 G-I). Thus, larger tumors (≥0.5 cm) in the presumed MEN2 cases were stronger stained for CgA than smaller tumors (≤0.2 cm) and the adjacent *in-situ* tumors (Table 1). Among 9 cases of sporadic cases, the youngest was 26 years old, and the oldest was a 66-year-old man (Table 2). Seven cases were primary thyroid tumors plus two cases of lymph node metastasis and one bone metastasis to the femur, respectively (Table 2). The primary tumors larger than 1.0 cm primary (≥1.0 cm) were moderately to strongly positive for CT in five cases (Case 1, 2, 7, 8, and 9). CgA immunostaining was diffusely, moderately to strongly stained in all six primary cases (Cases 1, 2, 3, 7, 8, and 9) while SPY staining was diffusely and strongly positive in three cases (Case 1, 7, and 8), and Case 9 was negatively stained for SPY (Table 2). The smallest sporadic MTC (0.5 cm × 0.3 cm), Case 4 was incidentally found in the lobectomy specimen performed for papillary thyroid carcinoma from a 42-year-old man (Table 2). This MTC was well-circumscribed and was located 2 cm away from the papillary carcinoma (2 cm × 2 cm × 1 cm); thus, this was a collision tumor. This MTC was partially, faintly positive for CT (about 70% of tumor cells), and patchy (about 10% of tumor cell nests) strongly for CgA and patchy (about 10% of tumor cell nests) weakly stained for SPY, respectively (Figure. 2A-C). The dysplastic-calcified lesion in the middle of the tumor was nonspecifically stained for CT, CgA, and SPY (Figure. 2A). Sporadic Case 1 from a 26-year-old man measuring 2.5 cm × 1.8 cm × 1.4 cm was diffusely moderately and strongly positive for CT and CgA, respectively, and diffusely and strongly positive for SPY (Figure. 2 D-F). Metastatic lymph node in Cases 2 and 6 was moderately stained for CT and CgA and weakly stained for SPY (Figure. 2 G-L). Case 5 of the metastatic bone lesion from a sporadic case showed fibrous non-osseous stroma embedded by trabecular tumor cells, which were weakly positive for CT, CgA, and SPY (Figure. 2J-L).

**TABLE 1:**
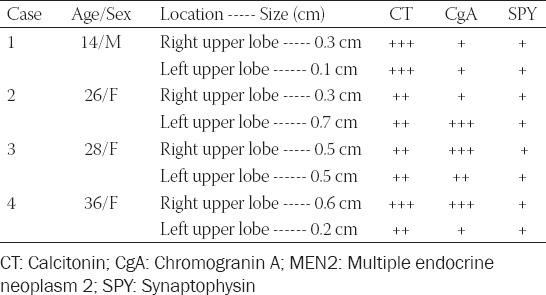
Immunohistochemical staining in presumed MEN2 and sporadic thyroid medullary carcinomas. Presumed MEN2 cases [4]

**TABLE 2:**
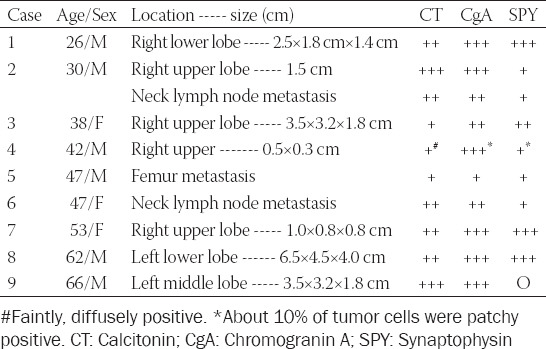
Sporadic cases [9]

## DISCUSSION

Among four cases of the presumed MEN2 cases, all four cases contained bilateral small MTC lesions in the upper one-third of the thyroid lobe, some of which (≤0.2 cm) were not grossly detected at the time of grossing the tissue. Case 3 contained 0.5 cm tumor in both lobes (Table 1). CgA staining was weakly stained in the tumors of <0.3 cm and moderately stained in the tumors of more than 0.5 cm in the presumed MEN cases (Table 1). Thus, in the presumed MEN2 cases, small tumors (≤0.3 cm) were less strongly stained for CgA compared to the larger tumors (≥0.5 cm), which were generally moderately and strongly stained for CgA (Table 1). SPY immunostaining was weakly positive in all four presumed MEN2 cases (Table 1). The less immunostaining for CgA and SPY at the peripheral microscopic lesion in the bilateral tumors in MEN2 cases also support the less aggressive biological nature of the early MTCs in MEN2 cases. Among seven sporadic primary cases, six cases (Cases 1, 2, 3, 7, 8, and 9) were as large as 1 cm (Table 2). Five of seven primary tumors in sporadic cases (≥1.0 cm) were diffusely, moderately to strongly positive for CT and strongly stained for CgA, respectively (Cases 1, 2, 7, 8, and 9) (Table 2). In sporadic cases, the smallest MTC, measuring 0.5 cm × 0.3 cm, was only partially (about 10% of tumor cells) strongly positive for CgA. In comparison, larger tumors ≥1.0 cm were diffusely, moderately to strongly positive for CgA (Table 2). Among six larger sporadic tumors ≥1.0 cm, three cases were strongly, and one case was moderately immunostained for SPY (Table 2). Therefore, larger METs in both MEN2 cases (≥0.5 cm) and sporadic cases (≥1.0 cm) were diffusely and positively stained for CgA (Table 1). From this study, CgA immunostaining may serve as a marker for larger tumors in MTCs. Larger tumors were more strongly positive for CgA than smaller tumors in both the presumed MEA2 and sporadic cases. Perhaps SPY immunostaining may also parallel to the sizes of the tumors. Since the introduction of direct DNA analysis of *RET* mutations, it is the primary method for screening family members at risk for hereditary MTCs [19-21]. *RET* mutation in MEN2A cases is of three levels: Level 3 is most aggressive cases and metastasizes in the 1^st^ year of life with mutations in *RET* codon 611, 618, 620, and 622; Level 2 is of a high risk of MTC with *RET* mutations in codons 609, 768,790, 804, and 891; and thyroidectomy is recommended for levels 3 and 2 patients before age 5, and level 1 is still a risk of developing MTC in *RET* mutations affecting the codons 609, 768, 790, 804, and 891 [20-22]. We do not have the *RET* mutation information in our archival cases, and at least Cases 2-4 may clinically fall in the level 1-2 category. The serum CT level is the basis for prophylactic timing thyroidectomy in family members who have inherited mutated *RET* alleles [21,22]. The elevating serum CT levels correspond to the enlarging METs [9,10,22]. Thus, serum CT level is the most important clinical marker for hereditary MTCs: normal levels----- <10 pg/ml, intermediate-----10 _~_ 100 pg/ml, and suspected level ---- >100 pg/ml [21,22]. Prognosis of MTCs depends on the stages of the tumors: Stage 1 with <2 cm is 100% alive 10 years after surgery, stage 2 with >2 cm still tumor localized in the thyroid is alive 93% after 10 years, stage 3 with local lymph node metastasis is 71% alive after surgery, and stage 4 with metastasis to skin, trachea, larynx, lungs, and bones is alive 21% in 10 years [9-11,22]. Sporadic Case 4, the smallest tumor (0.5 cm × 0.3 cm, Table 2) was partially, faintly positive for CT (about 70% of tumor cells) and was patchy (about 10% of tumor cell nests) strongly and weakly stained for CgA and SPY, respectively, with dysplastic calcification in the middle of the tumor, suggestive of the slow growth of the tumor (Table 2). Sporadic Case 4 was incidentally found in the thyroid lobectomy specimen operated for papillary thyroid carcinoma. Simultaneous occurrence of medullary and papillary thyroid carcinoma has been reported. MTC derives from the C-cells of the ultimobranchial body of the neural crest [23]. At the same time, the most common thyroid cancer is papillary thyroid carcinoma (70-80%), followed by follicular carcinoma (20%), both derive from the follicular cells of the endoderm [23-25]. The incidence of thyroid carcinoma increased about three folds from 1973 to 2002, attributable to the increased incidence of papillary carcinoma, likely due to the increasing histopathological diagnosis for this tumor [26], while there was no significant increase of incidence for follicular, medullary, and anaplastic carcinoma [26,27]. Anaplastic carcinoma usually transforms from well-differentiated thyroid carcinoma [26]. The majority of the reported papillary and medullary carcinomas were mixed papillary-medullary carcinomas [28-31]. At least 20 concurrent papillary and MTCs have been reported in the English literature [28-32]. Our sporadic medullary carcinoma, Case 4 was separate from the papillary carcinoma, and thus this was a collision tumor as also reported by the others [30,33]. The simultaneous thyroid double tumor is caused by point mutation of *RET* proto-oncogene [7,32]. However, one collision tumor reported by Dikbas *et al*. was negative for *RET* gene mutation [33]. Chromosomal rearrangement resulting in fusing RET protein appears to play an oncogenic role in 20% of papillary thyroid carcinomas [7,8,34]. One hundred twenty cases reported with bone metastasis among the 416 MTC cases (29%), 97% was non-osseous like the sporadic Case 5, and these cases followed poor prognosis [35]. CgA is the driving force for the biogenesis of secretory granules. It induces the budding of the trans-Golgi network membranes forming dense granules, thus influencing the pro-hormones transport into the secretory granules [36]. To release hormone, secretory granules make contact with the plasma membrane (docking) and fuse with the plasma membrane (exocytosis) [36,37]. After co-secreting CgA with each peptide hormone, CgA is recycled back to the new cycle of hormones secretion. At the same time, a part of it is secreted into the blood, thus modulates the peptide hormone secretory cycle [36-39]. CgA and SPY are co-located in the endocrine cells, and CgA occurs more basically in the endocrine cell cytoplasm, corresponding to the location of neurosecretory granules, while SPY immunostaining occurs more diffusely outside the secretory granules, corresponding to the diffuse distribution of synaptic vesicles (SVs) in the cytoplasm [40,41]. There is a correlation between serum CgA levels and endocrine tumor growths in several neuroendocrine tumors (NETs) [14,42,43]. Serum CgA levels were reportedly elevated in 100% of gastrinomas, 89% of pheochromocytomas, 80% of small intestinal carcinoids, 69% of non-functioning pancreatic NETs (Pan-NETs), and 50% of MTCs, in which tumor growth paralleled to serum CgA levels [42-44]. The baseline serum CgA levels of patients with MTC were 184 ng/ml, and the maximum serum level was 13,900 ng/ml compared to the baseline serum levels of control patients of about 100 ng/ml [43,44]. The serum CgA levels in ng/ml are more than 1000 times higher than that of serum CT levels in 10–100 pg/ml [20,21]. The serum CgA and pancreatic polypeptide levels increased more than 3-5 times above the baseline levels after a protein meal. The serum CgA levels of patients with MTC would be much higher after protein meal [42-45]. MTCs invade the vital neck organs and may not spread to the other distant organs when patients succumb to the tumor [1-6]. This relatively limited tumor spread may be a reason for not presenting highly elevated serum CgA levels in MTC patients [1,5,14,42,43]. The main study on serum CgA levels had been reported in the intestinal carcinoids: The midgut carcinoids occurring in the stomach, duodenum, small intestine presented largely elevated serum CgA levels, and the median survival of patients with serum CgA >5 mg was 33 months compared to 57 months in patients with serum CgA level <5 mg [14,39-43]. Thus, serum CgA levels paralleled the spread of gastrointestinal carcinoids [14,42,43]. There was also a correlation between serum CgA levels and tumor progression: Elevated serum CgA levels were reported in 83% of gastroenteropancreatic (GEP)-NETs tumors and elevated serum CgA levels were present in 100% of cases with liver metastasis [14,42,43]. In GEP-NETs, high serum CgA levels correlated with shorter survival and liver metastasis as reported in small intestinal NETs accompanied by up to 200 times above normal levels and up to 150 times higher MEN1 cases [14,42,43]. A sudden increase in serum CgA was accompanied by rapid tumor growth and short survival [42]. In Pan-NETs, both functioning and non-functioning tumors showed serum CgA levels at 60 _~_ 80 times the normal upper levels, particularly in Zollinger-Ellison syndrome in MEN1 cases with serum CgA levels being 80 _~_ 100 times higher than the normal upper levels [14,42,43]. So far, CgA is gaining acceptance as a serum marker of GEP-NETs and other NETs, including MTCs [14,16,42-44]. Among normal pancreatic islet cells, β-islet cells were weaker stained for CgA than the non-β-cells such as α, ϭ, and PP cells [16,36,45]. Among Pan-NETs, insulinomas were weaker stained for CgA than the non-β-cell tumors, including gastrinomas, glucagonomas, pancreatic polypeptidomas, and non-functioning Pan-NETs [14,16], and these non-β-cell Pan-NETs are more aggressive than insulinomas [14,16]. A collaborative study on serum CgA and tumor tissue CgA levels is warranted correlating the tumor sizes and locations with the serum CgA levels. The specific serum biomarker of MTCs is CT, and additional markers are serotonin, CgA, tryptase, and urinary markers are catecholamine and histamine [1,2]. The SVs, spheres of 40 nm in diameter, that constitute the central organelle to store and release classic neurotransmitters such as acetylcholine, norepinephrine, serotonin, GAVA, glycine, histamine, and glutamate, do not contain usual secretory granules [46,47]. SPY belongs to a family of related vesicle proteins present in small SVs, which includes Synaptotagmin (p65), SNAP-25, SNAP-receptor, Syntaxin, Rab3A, Synaptoporin, Pantophysin (SYPL1), Mitsugumin (SYPL2), Synaptogyrins 1-4(SNG 1-4), and others [46-50]. SPY was the first synaptic protein cloned and is the most abundant SV protein, accounting for about 10% of total SV proteins [46-49]. Presynaptic nerve terminals release neurotransmitters by SV endocytosis [47-50]. Previous molecular studies have hinted at several diverse roles for SPY in synaptic function, synapsis formation, biosynthesis, and endocytosis of SVs [48-50]. From this study, CgA and SPY appear to be involved in the MTC growth process, in which strong CgA and SPY immunostaining may support robust CT synthesis and secretion of CT, paralleling with aggressive growth in MTCs. However, SVs structure-function relationship with peptide hormone synthesis and secretion is still elusive [51,52].
